# Growth and Characterization of Ga_2_O_3_ for Power Nanodevices Using Metal Nanoparticle Catalysts

**DOI:** 10.3390/nano15151169

**Published:** 2025-07-29

**Authors:** Badriyah Alhalaili, Antony Joseph, Latifa Al-Hajji, Naser M. Ali, Sowmya Dean, Ahmad A. Al-Duweesh

**Affiliations:** 1 Nanotechnology Application Program, Energy and Building Research Center, Kuwait Institute for Scientific Research, Safat 13109, Kuwait; ajoseph@kisr.edu.kw (A.J.); lhajji@kisr.edu.kw (L.A.-H.); nmali@kisr.edu.kw (N.M.A.); sdean@kisr.edu.kw (S.D.); aduweesh@kisr.edu.kw (A.A.A.-D.); 2 American Romanian Academy of Arts and Sciences, Citrus Heights, CA 95616, USA

**Keywords:** *β*-Ga_2_O_3_, thermal oxidation, catalysts, silver, gold

## Abstract

A simple and inexpensive thermal oxidation process is used to grow *β*-Ga_2_O_3_ oxide (*β*-Ga_2_O_3_) thin films/nanorods on a *c*-plane (0001) sapphire substrate using Ag/Au catalysts. The effect of these catalysts on the growth mechanism of Ga_2_O_3_ was studied by different characterization techniques, including X-ray diffraction analysis (XRD), Scanning Electron Microscopy (SEM), and Energy Dispersive X-ray analysis (EDX). The XRD results of the grown Ga_2_O_3_ on a sapphire substrate show three sharp diffraction peaks located at 19.31°, 38.70° and 59.38° corresponding to the $\left(\bar{2}01\right),\;\left(\bar{4}02\right)$ and $\left(\bar{6}03\right)$ planes of *β*-Ga_2_O_3_. Field Emission Scanning Electron Microscope (FESEM) analysis showed the formation of longer and denser Ga_2_O_3_ nanowires at higher temperatures, especially in the presence of silver nanoparticles as catalysts.

## 1. Introduction

Gallium oxide (Ga_2_O_3_), a semiconductor with a broad bandgap of about 4.9 eV, has drawn a lot of interest because of its potential applications in ultraviolet, photodetection, high-power electronics and optoelectronics [1,2]. Its exceptional properties, such as excellent electron mobility, high breakdown voltage and outstanding thermal stability, make it an ideal candidate for high-temperature and high-voltage applications [3]. However, the performance of Ga_2_O_3_-based devices depends on the material’s crystal size, morphology and defect density. Therefore, understanding and controlling the growth mechanism of Ga_2_O_3_ is crucial for optimizing its properties and scaling up production for industrial purposes.

Several studies have been reported in the literature to investigate the growth mechanism and produce high-quality Ga_2_O_3_ nanostructures using various methods. Shi et al. [4] created Ga_2_O_3_ thin films using vacuum thermal evaporation and post-annealing in air at different temperatures. They observed an increase in the bandgap from 4.70 eV to 5.13 eV as the annealing temperature increased from 400 °C to 1100 °C. While this post-annealing procedure improves the quality of the material, it requires high temperatures, leading to higher energy costs and longer processing times. This may be expensive, especially for large-scale production. Recent research has explored the use of metal nanoparticles, such as gold and silver, as catalysts to enhance the formation of Ga_2_O_3_ nanostructures, with the aim of solving these drawbacks [5,6].

Catalysts such as Ag and Au significantly enhance the wettability of liquid gallium on substrates, leading to more oxidation and high-density formation of Ga_2_O_3_ nanostructures. Alhalaili et al. [7] demonstrated that using an Ag catalyst with photoelectrochemical etching on silicon surfaces significantly improved Ga wettability, resulting in homogeneous and dense development of Ga_2_O_3_, even in deep pores. Without the Ag catalyst, Ga formed irregular droplets causing non-uniform Ga_2_O_3_ production and partial oxidation. These catalysts are crucial for regulating the shape and quality of Ga_2_O_3_ nanostructures. In addition to increased Ga wettability, Ag and Au catalysts help promote oxygen molecule dissociation by lowering the activation energy for oxygen adsorption and dissociation on their surfaces, facilitating the reaction with gallium to form Ga_2_O_3_. This process involves the metal nanoparticles acting as oxygen reservoirs, where oxygen molecules adsorb onto the catalyst surface, dissociate into atomic oxygen and subsequently react with liquid gallium to nucleate Ga_2_O_3_ nanostructures [8,9].

Keerthana et al. [10] found that Ag-decorated *β*-Ga_2_O_3_ heterostructures have better photocatalytic activity for hydrogen evolution due to improved electron-hole pair separation. Woo et al. [11] developed an Ag/GaO_3_ bilayer electrode for near-ultraviolet light-emitting diodes (LEDs), which showed enhanced conductivity, stability and transparency, improving LED performance. Zhou et al. [12] studied the interstitial oxygen position in both Ag and Au, finding that while Au had a higher oxygen diffusivity, Ag had a higher oxygen concentration. These findings further support the role of Ag and Au as catalysts. The growth rate and quality of Ga_2_O_3_ can be influenced by this variation in oxygen dynamics, where Ag promotes the synthesis of Ga_2_O_3_ and Au facilitates faster oxidation.

This study investigates the growth of Ga_2_O_3_ nanostructures on sapphire substrates using Ag and Au nanoparticles as catalysts at 800 °C and 900 °C. Unlike previous research work that generally discussed catalyst effects, our work focuses on how temperature, catalyst type and catalyst concentration affect Ga_2_O_3_ morphology, crystallinity and composition. These variables were analysed with X-ray diffraction (XRD) and Field Emission Scanning Electron Microscope Energy Dispersive Spectroscopy (FESEM-EDS). The FESEM analysis reveals how different temperatures and catalyst conditions affect the formation of denser nanowires or nanosheets, aiding in optimizing properties for optoelectronics, sensors and high-power electronics.

The synthesis of Ga_2_O_3_ nanowires in the presence metal nanoparticles such as Ag [8,9] or Au [13] significantly influences developments in electronics and optoelectronics. These Ga_2_O_3_ nanowires have potential applications in FinFETs [14], UV detectors [9] and field-emitters [15]. Previous studies, such as those by Chunyang et al. [13], Xu et al. [14] and Tang et al. [15], have utilized Au nanoparticles as catalysts for β-Ga_2_O_3_ nanowire growth via metal-organic chemical vapor deposition (MOCVD) and demonstrated applications in FinFETs and field-emitters. In contrast, this work employs a cost-effective thermal evaporation method, which is simpler and more scalable than MOCVD, reducing energy costs and equipment complexity. Furthermore, we compare the efficacy of Ag and Au catalysts, revealing that Ag nanoparticles enhance the formation of denser and longer *β*-Ga_2_O_3_ nanowires due to higher oxygen solubility and diffusion, a novel aspect not extensively explored in prior studies. This systematic investigation of catalyst type, concentration, gallium amount and temperature provides critical insights for optimizing *β*-Ga_2_O_3_ nanostructures for applications in optoelectronics, sensors and high-power electronics.

## 2. Materials and Methods

### 2.1. β-Ga_2_O_3_ Thin Films/Nanorod Growth

**Figure 1:**
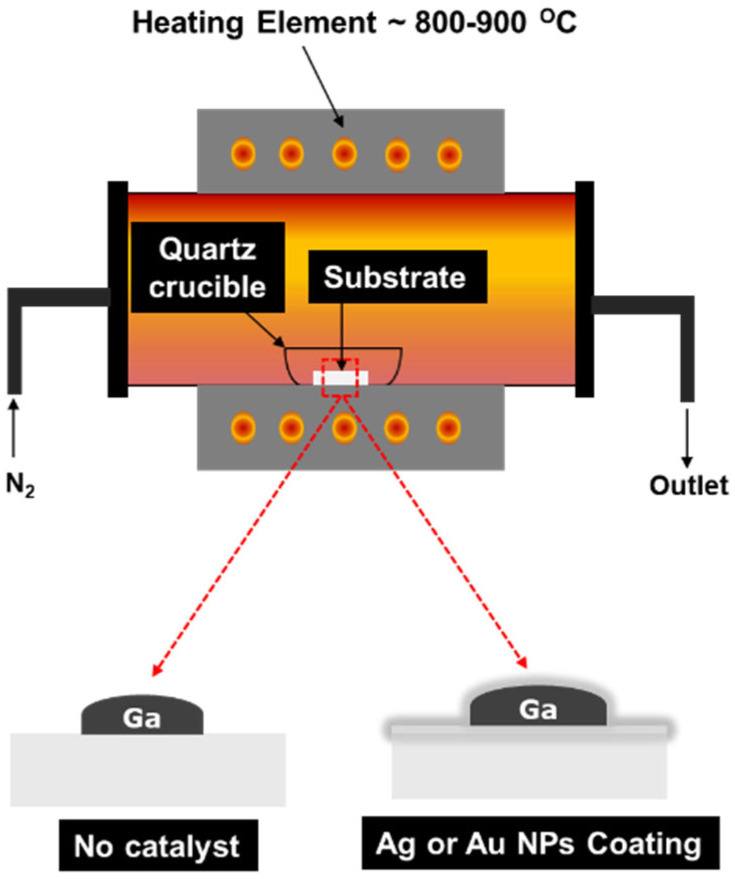
Graphical illustration of sample set-up inside the furnace used for growing and studying the morphology of Ga_2_O_3_ using different catalysts.

The thermal evaporation method was used to grow *β*-Ga_2_O_3_ thin films/nanorods on an Al_2_O_3_ substrate (100 × 0.9 mm, single-polished) with two different catalysts. This method is inexpensive and compatible with the preparation of a wide variety of nanomaterials. Silver and gold nanopowders (99.99%, 50–100 nm) obtained from US Research Nanomaterials, Inc. Corporation (Houston, TX, USA) were used as catalysts. The sapphire substrates were cleaned using acetone and ethyl alcohol, rinsed with deionised water and dried with a nitrogen gun. A total of 0.2 grams of gallium (Ga), with a purity of 99.99% obtained from Sigma Aldrich, St. Louis, MO, USA, was carefully dripped onto the polished surface of the sapphire substrate. After that, the catalyst nanopowder was added in varying amounts: 0, 5, 10 and 15 milligrams. For comparison, samples were grown under identical conditions on bare substrates. The samples were placed into a quartz crucible and then loaded into a Protherm laboratory tube furnace (model PTF 12/105/750) made in Turkey. This horizontal alumina tube furnace has a temperature range of up to 1200 °C, with a power of 3300 W and a current of 15 A. It operates at a frequency of 50–60 Hz and heating occurs in a 20 sccm nitrogen atmosphere. The oxygen required for Ga_2_O_3_ formation was sourced from residual oxygen present in the nitrogen atmosphere (20 sccm) within the tube furnace and from oxygen dissolved in the Ag and Au nanoparticles, which serve as oxygen reservoirs due to their high oxygen solubility [16,17]. An oxygen analyser (EO-W/1000, MTI Corporation, Richmond, CA, USA) was used to monitor the background oxygen concentration during the experiment. Figure 1 illustrates the sample set-up inside the furnace, which was used to grow gallium oxide on the alumina surface.

### 2.2. Characterisation Method

The surface morphological properties and elemental characterisation of the as-grown Ga_2_O_3_ thin films and nanorods were analysed by a Field Electron Scanning Electron microscope equipped with Energy-Dispersive X-ray spectroscopy (FESEM-EDX). X-ray diffraction (XRD) analysis was conducted to identify the crystal nature of the material formed using a Rigaku MiniFlex-600 diffractometer with Cu Kα (λ = 0.15406 nm; 20–40 kV, 2–15 mA) from 10° to 70° two-theta (2θ) at the temperature of 21° in step-scan mode with a scanning rate of 1°/min.

## 3. Results and Discussions

### 3.1. X-Ray Diffraction Analysis

**Figure 2:**
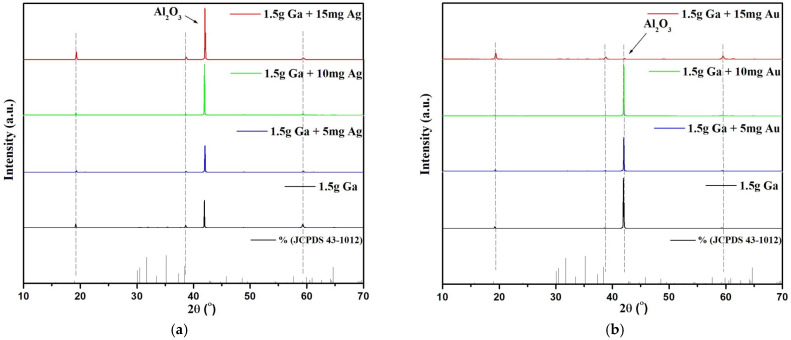
XRD pattern of *β*-Ga_2_O_3_ nanowires grown on the surface of sapphire at 800 °C: (**a**) Ag NP catalyst, (**b**) Au NP catalyst.

X-ray diffraction analysis was used to study the effect of different catalyst loadings during the preparation of Ga_2_O_3_ and the crystal quality of the thin films/nanorods grown on the sapphire substrate. The X-ray diffraction pattern (Figure 2) of gallium oxide crystals grown on sapphire surfaces at 800 °C with varying amounts of silver catalyst. The X-ray diffraction pattern clearly shows that the gallium oxide crystals grown on the surface of sapphire using Ag/Au as a catalyst are pure *β*-Ga_2_O_3_ without any other gallium oxide phases [18]. A preferential crystal growth in the $\left(\bar{2}01\right)$ direction can be observed with the increase in the amount of catalyst due to the preferred arrangement of oxygen atoms in the *β*-Ga_2_O_3_ $\left(\bar{2}01\right)$ plane that is equivalent to sapphire (0001) [19]. No other peaks corresponding to different gallium oxide phase configurations were seen in the XRD spectrum.

**Figure 3:**
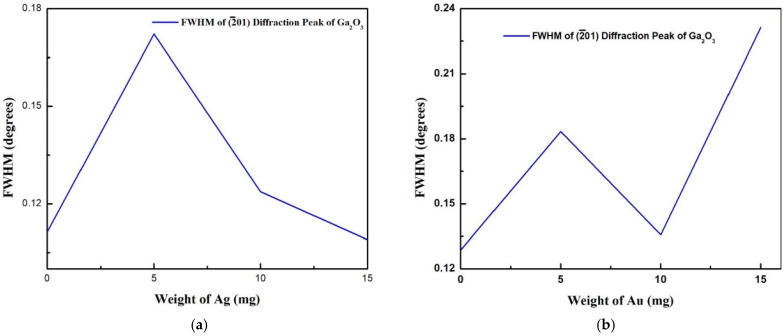
FWHM of $\left(\bar{2}01\right)$ diffraction peak of *β*-Ga_2_O_3_ with different catalyst loading at 800 °C: (**a**) Ag catalyst, (**b**) Au catalyst.

Figure 3 shows the variation of the full width at half maximum (FWHM) of the $\left(\bar{2}01\right)$ diffraction peak of *β*-Ga_2_O_3_ with different catalyst loading. For the Ag catalyst, as the amount of Ag increased from 5 mg to 15 mg, an increase in the intensity of the $\left(\bar{2}01\right)$ diffraction peak and a decrease in the FWHM were observed. However, no clear trend was observed in the case of the Au catalyst.

### 3.2. Effect of Temperature on the Growth of Ga_2_O_3_ by FE-SEM Analysis

The temperature of the reaction medium significantly influences the kinetics mechanism of Ga_2_O_3_ nanoparticle growth. The physical properties, such as the solubility and diffusivity of oxygen molecules on metal nanoparticles, change with temperature. Specifically, the oxygen diffusion coefficients of Ag and Au nanoparticles are crucial in determining the growth density and length of Ga_2_O_3_ nanostructures. As the temperature increases, the diffusivity of oxygen molecules on these metal nanoparticles also increases, thereby promoting the growth of Ga_2_O_3_ nanostructures [8,20]. Similarly, the solubility of oxygen molecules in metal nanoparticles increases with temperature. At higher temperatures, the surface energy of nanoparticles increases, causing increased diffusion of gas molecules at the nanoparticle surfaces. Thus, increasing the surface area of nanoparticle droplets will lead to increased oxygen adsorption and diffusivity. In addition, it increases the mobility, agglomeration and distribution of NPs [18], leading to an increase in oxygen absorption.

**Figure 4:**
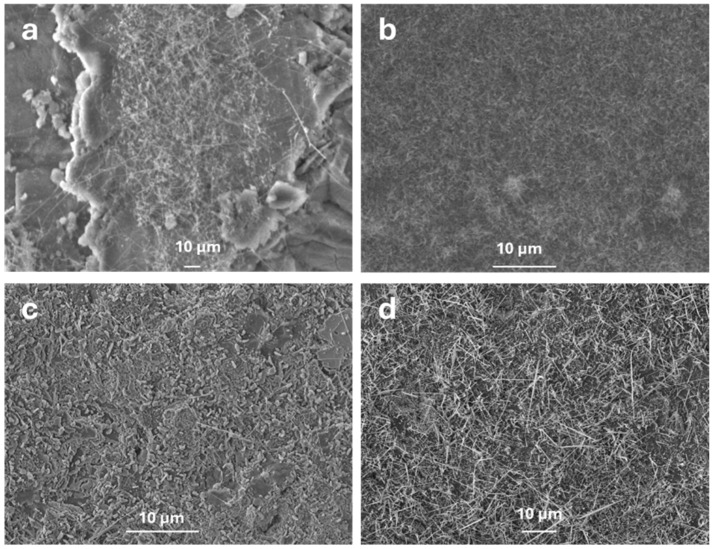
Effect of the oxidation temperature on the growth of Ga_2_O_3_ in the presence of Au NPs (**a**) at 800 °C; (**b**) at 900 °C and in the presence of Ag NPs (**c**) at 800 °C; (**d**) at 900 °C.

The effect of temperature on the growth mechanism of Ga_2_O_3_ was studied by conducting the thermal oxidation process at 800 °C and 900 °C in the presence of Ag and Au nanoparticles (50–100 nm) deposited on the surface of liquid gallium. For the first time, an enhancement in the growth of Ga_2_O_3_ nanowires was observed during the thermal oxidation process. These results are consistent with the previous studies [12,21], highlighting the impact of catalytic effects at elevated temperatures. Figure 4 shows the SEM images of Ga_2_O_3_ nanostructures grown in the presence of metal nanoparticles at 800 °C and 900 °C for a duration of 45 min. The SEM image shows the growth of nanocrystalline grains coated with short nanostructures at 800 °C, whereas longer, denser and thinner nanowires were formed at 900 °C. This is due to the increased oxygen solubility of metal catalysts (i.e., Au or Ag) above its melting point. Even though the solubility of oxygen gases usually decreases in liquids with increasing temperature, for a few gases in some solvents and temperature ranges, solubility increases with an increase in temperature [22,23,24]. Silver exhibits high oxygen solubility at elevated temperatures, as reported by Ramanarayanan et al. [16], which enhances the availability of oxygen for Ga_2_O_3_ nucleation, contributing to the formation of denser and longer nanowires.

The variability in size and direction of *β*-Ga_2_O_3_ nanostructures observed in Figure 4 can be attributed to the self-diffusion of metal nanoparticles, leading to non-uniform catalyst distribution. To improve growth uniformity, techniques such as substrate pre-patterning via lithography or template-assisted deposition can be employed to control nanoparticle placement, as demonstrated in prior work with Ag-patterned quartz [8]. Additionally, optimizing substrate surface roughness and implementing precise control of oxygen flow during thermal evaporation could enhance directional growth and uniformity. These strategies will be investigated in future studies to achieve more consistent nanowire morphology.

### 3.3. Effect of Gallium Concentration on the Growth of Ga_2_O_3_ by FE-SEM

**Figure 5:**
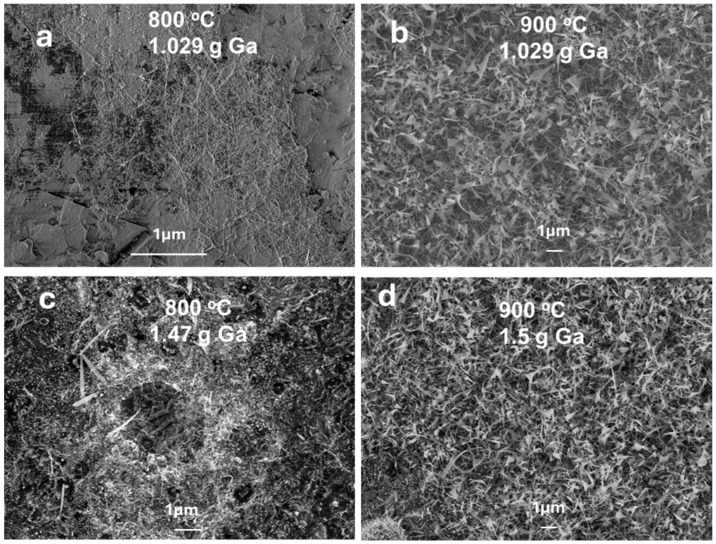
FESEM Images of Ga_2_O_3_ nanostructures grown using Au. (**a**,**b**) Low Ga concentration at 800 °C and 900 °C. (**c**,**d**) High Ga concentration at 800 °C and 900 °C.

**Figure 6:**
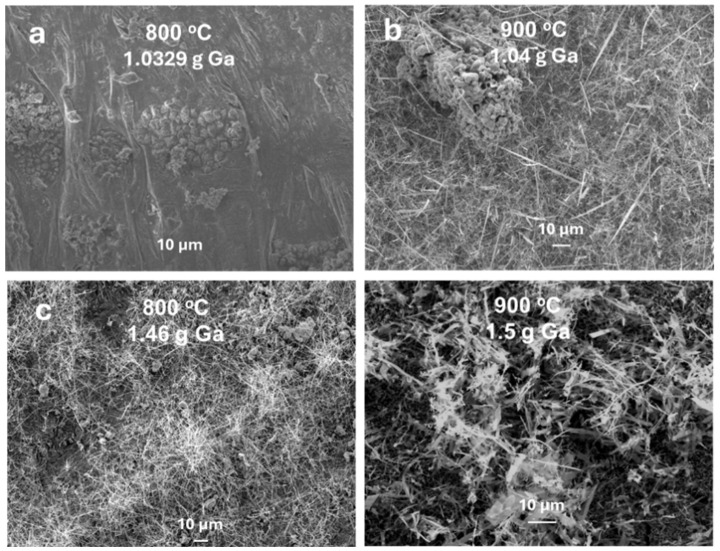
FESEM Images of Ga_2_O_3_ nanostructure grown using Ag. (**a**,**b**) Low Ga concentration at 800 °C and 900 °C. (**c**,**d**) High Ga concentration at 800 °C and 900 °C.

Gallium concentration is one of the critical factors controlling the growth of Ga_2_O_3_ nanostructures. Figure 5 and Figure 6 represent FESEM images of Ga_2_O_3_ nanostructure grown using Au NPs and Ag NPs, respectively. A common observation is that as the concentration of gallium increases, the density of Ga_2_O_3_ nanostructures increases. This is attributed to the higher tendency of gallium to react with oxygen. In addition, the presence of Ag or Au nanoparticles enhances the absorption capacity, leading to high-density nanostructures [16].

As the diffusion length of Ga increases with temperature, more Ga atoms diffuse to metal nanoparticles [25]. Consequently, liquid Ga extracts oxygen from the metal nanoparticles, which act as a reservoir of more oxygen at the interface, allowing it to react with Ga. This leads to the nucleation of Ga_2_O_3_ with high density at 800 °C. However, at 900 °C, Ga_2_O_3_ nanowires or nanosheets continue to grow as the diffusivity and solubility of oxygen molecules increase with temperature. This results in denser and longer nanowire (Figure 5 and Figure 6d) due to the supersaturation of various species. Annealing at 800 °C causes the metal catalysts to react with more residual oxygen molecules in the chamber, increasing the concentration of O_2_ in the metal [25]. At all temperatures, the oxidation enthalpy of Ga is much higher than that of Ag. Nucleation of Ga_2_O_3_ is expected to initiate on the metal nanoparticle surface, as observed in Figure 5a and Figure 6a for Au and Ag catalysts, respectively, at lower gallium concentrations and temperatures.

The presence of Ag or Au on the bare quartz and Ga enhances nucleation of Ga_2_O_3_. The kinetics and growth mechanism have been explained earlier in details [9]. Due to the self-diffusion of metal nanoparticles, more oxygen molecules will enhance the dissolution of gallium suboxide (Ga_2_O) in the liquid metal nanoparticles and deposit Gallium (III) oxide (Ga_2_O_3_) through crystal nucleation on the bare quartz by a vapor–liquid–solid (VLS) growth mechanism to increase the synthesis of Ga_2_O_3_ nanostructures [26].

The incorporation of Ag or Au catalysts enhances the growth process to a denser and longer *β*-Ga_2_O_3_ nanowires or nanostructures due to several factors that increase oxygen molecules in the system. For example, the solubility of O_2_ in Ag and Au [27] is much higher than the solubility of oxygen in liquid Ga molecules [28]. Furthermore, the oxygen molecules solubility in Ag nanoparticles are significantly increased by increasing the temperature at fixed pressure, leading to more oxygen molecules attraction to increase the growth of Ga_2_O_3_ nanostructures. Interestingly, the Ga atoms enthalpy is much more negative than that of the oxidation of Ag or Au, at all temperatures. This causes a constant nucleation of Ga_2_O_3_ nanostructures to occur on Ag-Ga-O liquid mixture, where Ga strips the dissolved O_2_ away from Ag nanoparticles liquid islands at the higher temperature.

**Figure 7:**
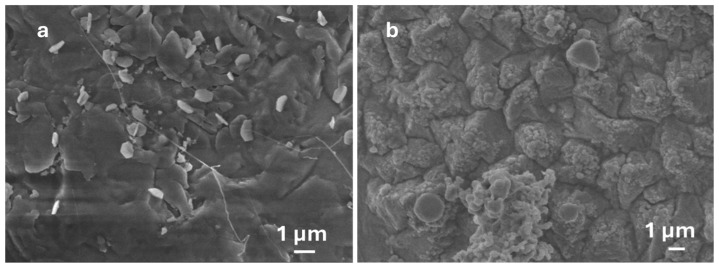
FESEM image of Ga_2_O_3_ nucleation grown using Ga is ~1.03 g and (**a**) 10 mg Au NPs and (**b**) Ag NPs at 800 °C.

At 800 °C, oxygen’s solubility in Ag nanoparticles is highly increased, leading to Ga_2_O_3_ nucleation as shown in Figure 7. However, at a higher temperature (1000 °C), the growth mechanism saturates when reaching equilibrium phase of AgGaO_x_ (Ag-Ga-O phase diagram No. 209084) [29]. Consequently, a constant amount of Ga_2_O and O_2_ in the presence of Ag nanoparticles would raise the growth of Ga_2_O_3_.

**Figure 8:**
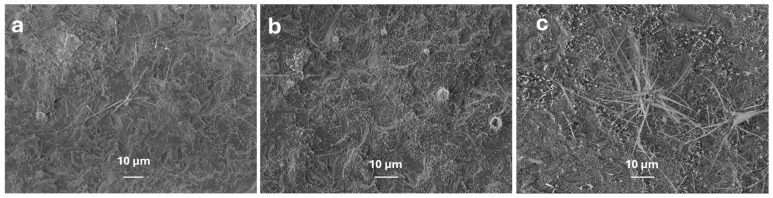
FESEM images of Ga_2_O_3_ nanostructures grown in the presence of 10 mg of Ag NPs at 800 °C. (**a**) 0.5 g of Ga; (**b**) 1.0 g of Ga; (**c**) 1.5 g of Ga.

Figure 8 shows the effect of various gallium concentrations at 800 °C in the presence of 10 mg of silver (Ag) nanoparticles. As the gallium concentration increases, the Ga_2_O_3_ nanowires becomes denser and thicker. This suggests that while the oxidation of metal nanoparticles enhances their attraction to oxygen molecules, a low concentration of gallium results in a lower density of Ga_2_O_3_ nanowires or nanosheets as previously observed by Yuan et al. [30].

**Figure 9:**
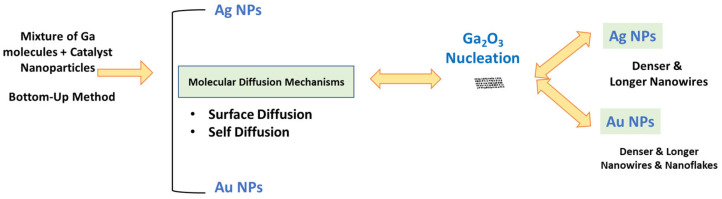
A schematic that summarizes the growth mechanism to obtain denser and longer Ga_2_O_3_ nanostructures in the presence of metal nanoparticles catalyst such as Au NPs or Ag NPs.

Different diffusion mechanisms, influenced by metal nanoparticles such as Ag and Au NPs, facilitate Ga_2_O_3_ nanostructures growth. Self-diffusion affects nanoparticle distribution, with Ag NP surface diffusion exhibiting the lowest activation energy (0.39 eV) compared to grain boundary (0.99 eV) and volume diffusion (1.98 eV) [31], allowing rapid, unconstrained movement and surface distribution. As temperature rises, surface diffusion increases, spreading more nanoparticles and enhancing Ga_2_O_3_ nanostructure growth [32,33]. Figure 9 summarizes how metal nanoparticle catalysts yield denser and longer Ga_2_O_3_ nanostructures.

### 3.4. Effect of Catalyst Selection and Concentration on the Growth of Ga_2_O_3_ by FE-SEM Analysis

**Figure 10:**
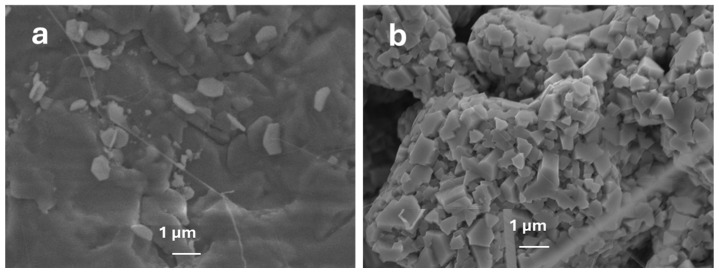
FESEM images of Ga_2_O_3_ nanostructures grown at 800 °C with 1.5 g of Ga and 5 mg of catalyst. (**a**) Au NPs. (**b**) Ag NPs.

The ideal catalyst metal nanoparticles play a crucial role in enhancing the growth of Ga_2_O_3_ nanostructures. Figure 10 shows the growth of Ga_2_O_3_ at 800 °C with two different catalysts, Ag and Au nanoparticles, under identical conditions. A sheet-like continuous morphology was observed for the Ga_2_O_3_ sample grown with Au nanoparticles, whereas much denser and thicker crystalline structures were observed for samples grown with Ag nanoparticles. Silver’s higher oxygen solubility than gold makes it an effective catalyst for the growth of Ga_2_O_3_ nanostructures. In addition, silver has the lowest activation energy for surface diffusion (0.39 eV) compared to the activation energy of grain boundary diffusion (0.99 eV) and volume diffusion (1.98 eV) [12]. Hence, silver nanoparticles can interchange easily with no restriction when distributing at the surface. At low activation energy and high diffusion rate, the flux of atoms increases as the concentration gradient and temperature increase. As the temperature increases, the density of metal nanoparticles also increases [34]. However, grain boundary and surface diffusion are both expected in the oxidation process. Surface diffusion can be further increased as the duration of thermal annealing increases at a fixed temperature [32,33]. The final morphology of the Ga_2_O_3_ nanoparticles is determined by the shape and size of the initial Ga_2_O_3_ crystal nucleation sites [17,35].

**Figure 11:**
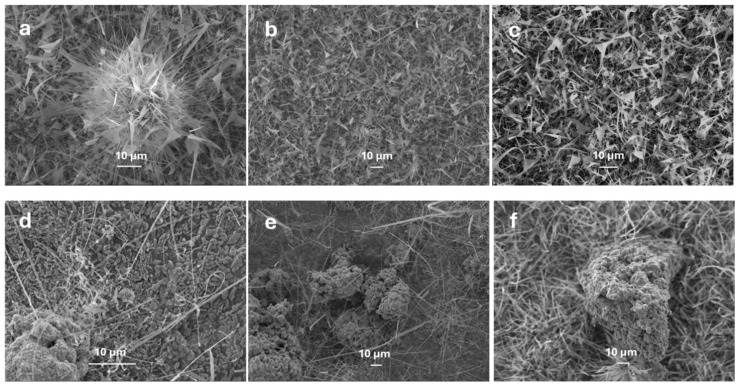
FESEM images of Ga_2_O_3_ nanostructures grown at 900 °C with 1.5 g of Ga and different catalyst concentrations. (**a**) 5 mg of Au, (**b**) 10 mg of Au, (**c**) 15 mg of Au, (**d**) 5 mg of Ag, (**e**) 10 mg of Ag and (**f**) 15 mg of Ag.

To better understand the kinetics and mechanism of interaction between metal nanoparticles (Ag and Au) and oxygen molecules, FESEM images of Ga_2_O_3_ nanostructures grown at 900 °C and 1.5 g of gallium with different amounts of catalysts (5, 10 and 15 mg) were analysed (Figure 11). Ga_2_O_3_ samples grown in the presence of Au exhibited more nanosheet-like textures, whereas Ag nanoparticles change the morphology of Ga_2_O_3_ nanostructures and form longer and denser nanowires. Our research team was able to achieve enhanced nanowire growth while using Ag as a catalyst due to its superior oxygen diffusion capability compared to other catalysts at higher temperatures. The oxygen diffusion coefficient of silver nanoparticles is much higher than Au [12], making Ag the best catalyst for enhancing the growth density and length of Ga_2_O_3_ nanowires. Although the diffusivity of oxygen molecules in Au nanoparticles is higher than in Ag nanoparticles, the oxygen molecule concentration in silver nanoparticles is higher than in gold nanoparticles [12]. This is due to the transformation of interstitial sites of oxygen molecules into O-Ag oxide, reducing the possibility of oxygen diffusion in silver nanoparticles, reducing the diffusion of oxygen, whereas gold nanoparticles are not easily transformed into O-Au oxide, allowing for higher oxygen diffusion in Au. Additionally, as the metal nanoparticle concentration increased, higher-density and longer Ga_2_O_3_ nanowires were formed (Figure 11). This can be primarily attributed to the increased availability of oxygen due to the presence of more metal nanoparticles.

The growth of *β*-Ga_2_O_3_ nanostructures using Ag and Au catalysts exhibits distinct differences. Ag nanoparticles promote the formation of denser and longer nanowires, while Au nanoparticles favor nanosheet-like structures (Figure 10 and Figure 11). This is attributed to Ag’s higher oxygen solubility and diffusion coefficient compared to Au [12], leading to enhanced nucleation and growth of *β*-Ga_2_O_3_ nanowires. XRD analysis (Figure 2) confirms that Ag-catalyzed samples exhibit sharper diffraction peaks, indicating superior crystal quality. Additionally, Ag’s lower activation energy for surface diffusion (0.39 eV compared to 0.99 eV for Au [31]) facilitates more uniform nanoparticle distribution, improving growth uniformity and ease of control. Therefore, Ag is the preferred catalyst for achieving high-quality, uniform *β*-Ga_2_O_3_ nanowires.

### 3.5. Elemental Composition of Ga_2_O_3_ by EDS Analysis

**Figure 12:**
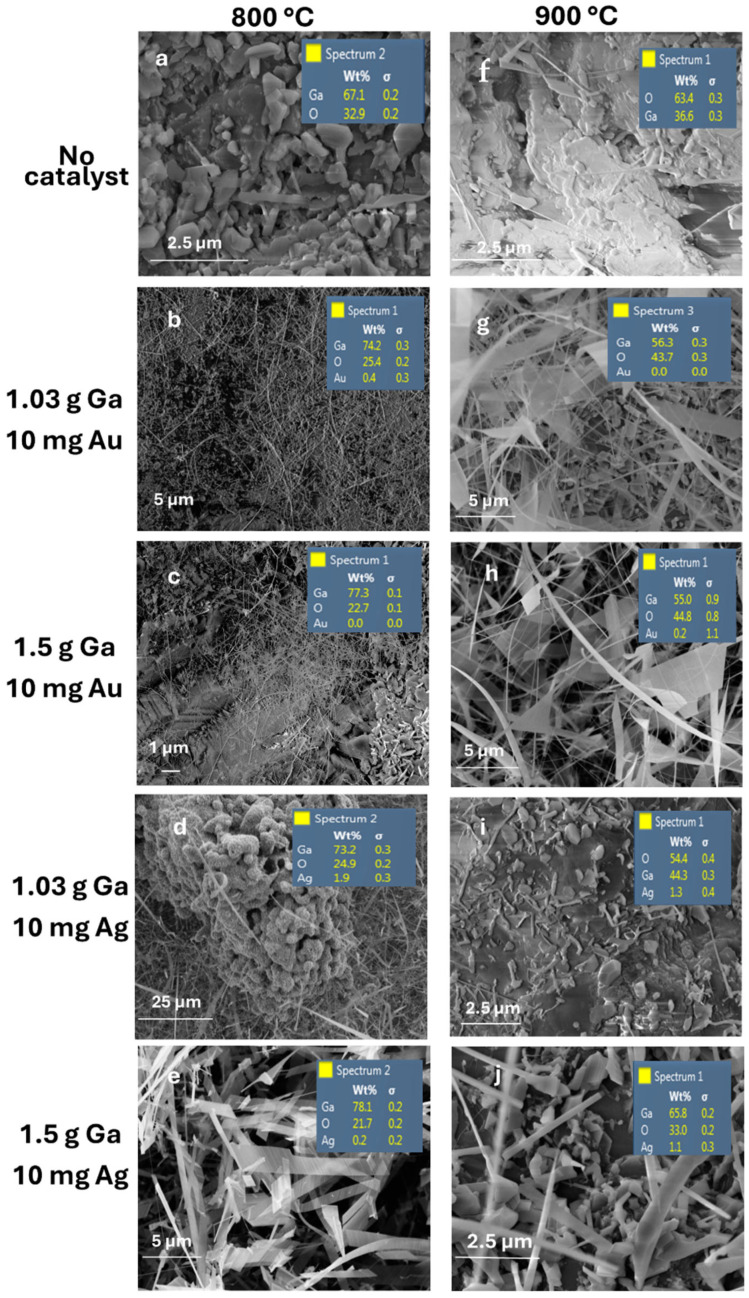
FESEM-EDS images of Ga_2_O_3_ nanostructure grown using Au NPs and Ag NPs. The left column at 800 °C and the right column at 900 °C; (**a**,**f**) are without catalyst; (**b**,**g**) shows Ga_2_O_3_ nanostructure grown using Ga is ~1.03 g and 10 mg Au NPs; (**c**,**h**) shows Ga_2_O_3_ nanostructure grown using Ga is ~1.5 g and 10 mg Au NPs; (**d**,**i**) show Ga_2_O_3_ nanostructure grown using Ga is ~1.03 g and 10 mg Ag NPs; (**e**,**j**) show Ga_2_O_3_ nanostructure grown using Ga is ~1.5 g and 10 mg Ag NPs.

Energy Dispersive Spectroscopy (EDS) was used to perform elemental and chemical microanalysis on the samples. Figure 12 presents the FESEM-EDX spectra of Ga_2_O_3_ nanostructures grown on sapphire substrates at 800 °C and 900 °C, with and without Au or Ag catalysts, corresponding to the elemental compositions listed in Table 1. The spectra confirm higher oxygen concentrations at elevated temperatures and in the presence of catalysts, consistent with increased oxygen solubility in liquid gallium and metal nanoparticles [16,24,25].

Compared to previous studies [36,37,38], we were able to create longer and denser Ga_2_O_3_ nanowires (Table 2). A careful analysis of the length and morphology of Ga_2_O_3_ nanowires grown using Au and Ag catalysts revealed that only Ag nanoparticles formed longer nanowires.

Interestingly, the percentage of oxygen absorption is much higher in both catalysts. However, the morphology of Ga_2_O_3_ has exposed major differences in the growth shape and direction of Ga_2_O_3_. Ag NP catalysts form denser and longer distribution of Ga_2_O_3_ nanowires only. However, Au NP catalysts forms denser nanowires and nanoflakes as well. Consequently, the presence of Ag NPs shows a better growth process rather than the Au NP catalyst in terms of growth uniformity and ease of control.

More investigation is required to evaluate the unintentional residual impurities of Ga_2_O_3_ nanowires with catalyst Au and Ag during their growth process. The influence of these impurities on device performance also requires further study. Some procedural challenges for the growth of Ga_2_O_3_ nanostructures also need to be explained. For instance, more directional Ga_2_O_3_ nanowire growth is highly desirable and the nanowires should also be more uniform in size and length. Finally, techniques to pattern and etch the Ga_2_O_3_ nanowires would be highly valuable.

A STEM image of a bright field (BF) and high-angle annular dark-field (HAADF) of a Ga_2_O_3_ nanowire grown at 1000 °C on uncoated quartz and on coated with 5 nm Ag thin film [9]. The bright field showed dark crystalline NPs due to diffraction contrast. Meanwhile, HAADF showed much brighter nanoparticles due to the z-contrast, suggesting that the presence of significant larger atomic mass and density than the adjacent *β*-Ga_2_O_3_, which points to Ag NPs. These Ag nanoparticles assist to synthesize *β*-Ga_2_O_3_ nanowires during the growth process and will be remain within the crystal lattice, exposing their size in the range of 1–5 nm [9] and it will be difficult to remove them.

Residual Ag and Au nanoparticles, ranging from 1–5 nm, are incorporated into the *β*-Ga_2_O_3_ nanowire lattice, as observed in STEM images. These impurities can enhance morphological, electrical and optical properties, such as bandgap and photoconductance, due to their catalytic role [9]. However, they may also introduce defects that could impact device performance, necessitating further investigation to quantify their concentration and effects. Techniques such as selective chemical etching or post-annealing in an inert atmosphere could be explored to minimize residual catalysts and improve nanowire purity.

## 4. Conclusions

Ga_2_O_3_ nanostructures grown on sapphire substrates using different catalysts (Au or Ag nanoparticles) were studied using X-ray diffraction analysis and FESEM-EDS to understand the growth mechanism, kinetics, and morphology of the nanostructures. The influence of different factors such as temperature, nature of the catalyst and concentration of the catalyst and Ga on the growth of Ga_2_O_3_ nanostructures were also studied using FESEM. The FESEM analysis showed the formation of more nucleation of Ga_2_O_3_ nanostructures at 800 °C, whereas much denser and longer nanowires and nanosheets were formed at 900 °C. The XRD results of the grown Ga_2_O_3_ on sapphire substrate show three sharp diffraction peaks located at 19.31°, 38.70° and 59.38° corresponding to $\left(\bar{2}01\right),\;\left(\bar{4}02\right)$ and $\left(\bar{6}03\right)$ planes of *β*-Ga_2_O_3_ (JCPDS No. 43-1012). A high atomic percentage of oxygen in Ga_2_O_3_ nanostructures grown on sapphire surfaces was detected by EDS in samples exposed to high temperatures. This is due to the increased oxygen solubility in liquid gallium and metal nanoparticles, as well as the effects of self-diffusion. Using silver and gold as catalysts, we obtained *β*-Ga_2_O_3_ nanowires thinner than those grown with other catalysts, such as Ni [37], Pt [40] or Fe [41], demonstrating the superior control over nanowire morphology offered by Ag and Au nanoparticles. Ag nanoparticles, as a catalyst for Ga_2_O_3_ nanowire growth, lead to denser nanowires, while Au nanoparticles result in more nanosheet-like structures of Ga_2_O_3_. With these morphology and structural characteristics, the proposed technique shows great potential for applications in optoelectronic devices, sensors and high-power electronics.

## Figures and Tables

**Table 1:** The elemental composition of Ga_2_O_3_ nanostructures grown on sapphire substrates at 800 °C or 900 °C. (**a**) without a catalyst; (**b**) with Au or Ag catalyst.

Temperatures	800 °C					900 °C			
(**a**)									
Elemental compositions (at. %)	Ga		O		Ga			O	
No catalyst	32.9		67.1		36.6			63.4	
(**b**)									
Elemental Composition (at. %)	Ga	O	Ag/Au		Ga		O	Ag/Au	
1.03 g Ga and 10 mg Au	74.2	25.4	0.4		56.3		43.7	0.0	
1.5 g Ga and 10 mg Ag u	77.3	22.7	0.0		55.0		44.8	0.2	
1.03 g Ga and 10 mg Au	73.2	24.9	1.9		54.4		44.3	1.3	
1.5 g Ga and 10 mg Ag	78.1	21.7	0.2		65.8		33.0	1.1	

**Table 2:** The average diameter, length and morphology of the nanowires were measured for 10 samples and the results were compared with those reported in the literature based on the different catalysts, techniques and substrates used.

Catalyst		Technique	Substrate	Average Nanostructures	
				Diameter	Length
Experimental Results (this work)					
**Au**		Oxidation	Sapphire	2–4 µm	15–25 µm
**Ag**				0.5–1.0 µm	>20 µm
**NPs**	**Ref.**	Reported Results			
**Free-Catalyst**	**[38]**	CVD	Ga/Ga_2_O_3_ mixture and O_2_	40–70 nm	10–20 µm
	**[37]**		Si/SiO_2_ & Al_2_O_3_	50-75 nm	>10 µm
**Ag NPs**	**[26]**	Sol-gel and VLS	GaAs	18 to 30 nm	several 10s of nm to a few 100 µm
**Au NPs**	**[39]**	Thermal-VLS	Au pre-treated on Si	34 nm	20 to 60 nm

## Data Availability

The raw data supporting the conclusions of this article will be made available by the authors on request.

## Abbreviations

The following abbreviations are used in this manuscript:

A	Ampere
Ag	Silver
Al_2_O_3_	Alumina
Au	Gold
Cu	Copper
EDX	Energy Dispersive X-ray
eV	Electron volt
FE-SEM	Field Emission Scanning Electron Microscope
FWHM	Full width at half maximum
Ga	Gallium
Ga_2_O_3_	Gallium oxide
Hz	Hertz
JCPDS	Joint Committee on Powder Diffraction Standards
LEDs	Light emitting diodes
NPs	Nanoparticles
O	Oxygen
Sccm	Standard cubic centimeters per minute
W	Watts
XRD	X-ray diffraction
λ	Lambda
